# Magnetic resonance imaging provides additional utility in the preoperative cartilage assessment of patients undergoing medial unicompartmental knee arthroplasty

**DOI:** 10.1002/ksa.12611

**Published:** 2025-02-10

**Authors:** Mei Lin Tay, Scott M. Bolam, Tyler Campbell, Laura Hill, Lydia Lin, Hayley Wong, David Dow, Jacob T. Munro, Simon W. Young, A. Paul Monk

**Affiliations:** ^1^ Department of Surgery, Faculty of Medical and Health Sciences (FMHS) University of Auckland Auckland New Zealand; ^2^ Department of Orthopaedic Surgery North Shore Hospital Auckland New Zealand; ^3^ Department of Orthopaedic Surgery Auckland City Hospital Auckland New Zealand; ^4^ Auckland Bioengineering Institute University of Auckland Auckland New Zealand

**Keywords:** MRI, patient selection, radiographic scores, unicompartmental knee arthroplasty

## Abstract

**Purpose:**

For unicompartmental knee arthroplasty (UKA), patient selection using correct indications can optimise postsurgical outcomes. The current gold standard for assessing eligibility is with radiographs; however, magnetic resonance imaging (MRI) may allow for more accurate assessments of cartilage damage. This study aimed to evaluate the utility of MRI for preoperative assessment of medial UKA patients by (1) comparing osteoarthritis severity of the medial, lateral and patellofemoral (PF) compartments when assessed using MRI compared with standard radiographs, and (2) investigating associations of these two assessments with postoperative clinical outcomes.

**Methods:**

This study had ethical approval. A retrospective review was performed for 88 primary medial UKA between 1 January 2017 and 31 December 2021. The main outcome measures were preoperative cartilage loss and patient‐reported clinical outcomes. Preoperative cartilage loss was recorded using the International Cartilage Repair Society (ICRS) classification using MRI, and Kellgren–Lawrence (K–L) scores from radiographs. Patient‐reported clinical outcomes were measured using preop, early (6‐week) and late (1‐ or 2‐year) Oxford Knee Score (OKS) change scores.

**Results:**

The use of MRI has improved accuracy over radiographs. In the medial compartment, 37 (44%) patients had less severe radiographic K–L scores (1–3); however, all patients had the most severe MRI ICRS scores (4). For patients with mild K–L scores (0 and 1), 20 (43%) and 7 (78%) patients had more severe ICRS scores (3 and 4) within their lateral and PF compartments, respectively. No associations were found between ICRS or K–L scores and OKS for any compartments.

**Conclusions:**

Assessment of medial cartilage thickness loss using MRI provides additional utility over standard radiographs in preoperative assessments of medial UKA patients. However, evidence of disease in the PF compartment assessed using MRI should not be considered a contraindication for UKA.

**Level of Evidence:**

Level III, retrospective cohort study.

**Abbreviations**
ACL: anterior cruciate ligament
AP: anterioposterior
BME: bone marrow oedema
BMI: body mass index
ICRS: International Cartilage Repair Society
K–L: Kellgren–Lawrence
MCL: medial collateral ligament
ML: mediolateral
MRI: magnetic resonance imaging
OA: osteoarthritis
OKS: Oxford Knee Scores
PF: patellofemoral
UKA: unicompartmental knee arthroplasty

## INTRODUCTION

The selection of patients with correct indications is important to optimise the success and longevity of unicompartmental knee arthroplasty (UKA) [19]. Accepted surgical indications for medial UKA are anteromedial bone‐on‐bone osteoarthritis (OA), full‐thickness cartilage in the lateral compartment and functionally intact medial collateral ligament (MCL) and anterior cruciate ligaments (ACL) [3, 9, 10]. Additionally, and particularly for the Oxford UKA, evidence of patellofemoral (PF) OA may not be considered a contraindication if the PF joint does not have full‐thickness cartilage loss within the lateral facet or trochlea, with any eburnation, grooving or subluxation [9, 10].

The traditional gold standard for assessing these features is with preoperative anteroposterior and mediolateral radiographs, which provide reliable sensitivity and specificity for experienced UKA surgeons [11, 13]. More recently, the use of magnetic resonance imaging (MRI) to provide higher accuracy in the assessment of the joint has become commonplace [12]. The high tissue contrast obtained with MRI provides improved resolution to delineate partial‐thickness cartilage defects and ACL or MCL deficiencies, compared with standard radiographs [13]. Specifically for medial UKA, MRI is useful for checking the integrity of the lateral and PF compartments and the ACL. Furthermore, MRI has the ability to detect additional structural abnormalities not visible on radiographs, such as subchondral bone marrow oedema (BME) [6] but may have the potential to affect clinical outcomes [1, 15, 30].

Although MRI can provide additional resolution compared to radiographs alone, some have suggested that it may be too sensitive for preoperative assessment of UKA patients [20, 23]. This could adversely lead to unnecessary costs or over‐utilisation of total knee arthroplasty for patients who would otherwise have good clinical outcomes with UKA. There is a need to better understand the perceived benefits and need of MRI for UKA patient assessment. There is currently no consensus on the most appropriate classification system of cartilage damage severity using MRI [23]. The International Cartilage Regeneration & Joint Preservation Society (ICRS) classification, initially developed for use in arthroscopy, is considered easily transferrable for MRI evaluation [7]. The ICRS MRI lesion classification is used here to evaluate the utility of MRI for preoperative assessment of medial UKA patients. The main aim of this study was to assess the benefits offered by MRI for preoperative cartilage assessment of UKA patients compared with standard radiographs by (1) comparing OA severity of the medial, lateral and PF compartments when assessed using MRI compared with standard radiographs, and (2) investigating associations of these two assessments with postoperative clinical outcomes.

## METHODS

### Patients and ethics

**Figure 1:**
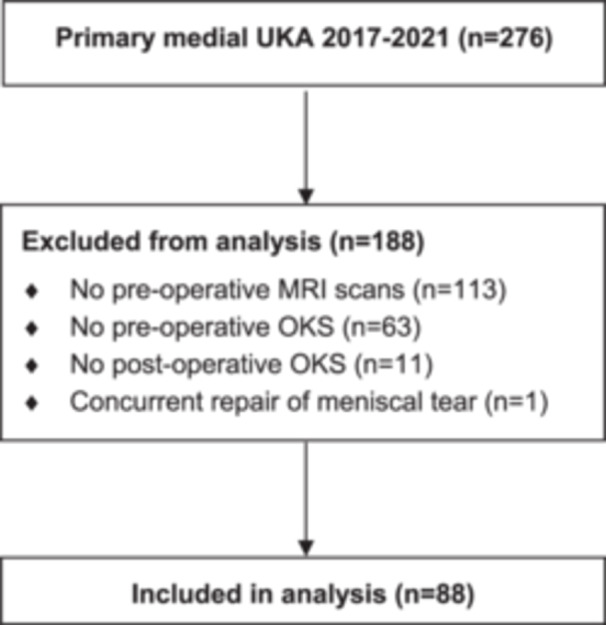
Flowchart of inclusion and exclusion for analysis of patients undergoing primary medial UKA at two tertiary centres between 2017 and 2021. MRI, magnetic resonance imaging; OKS, Oxford Knee Score; UKA, unicompartmental knee arthroplasty.

This study was carried out with ethics approval from the Auckland Health Research Ethics Committee, AH2545. A retrospective review of patients undergoing primary medial UKA surgery at two tertiary centres between 1 January 2017 and 31 December 2021 was performed. Inclusion criteria were (1) a primary medial UKA during the study period, (2) a primary diagnosis of OA, (3) preoperative radiographs and MRI scans, (4) pre‐ and postoperative Oxford Knee Score (OKS) responses and (5) minimum 1‐year follow‐up following UKA. Patients without preoperative MRI scans (*n *= 113) or OKS (*n* = 74) were excluded from the study (Figure 1). One further patient with a concurrent lateral meniscal tear that was repaired intraoperatively was also excluded. The study included a total of 88 cases (85 patients) from 11 surgeons with a mean follow‐up of 3.2 years ± 1.1 (SD, standard deviation; range 1.0–5.4). Patients were 57% male, with a mean age of 64.1 ± 8.8 (46–84) years and a mean body mass index of 29.7 ± 5.3 (18–44; Table 1). Implants used were Restoris MCK (Stryker Orthopaedics; 56 cases, 64%), Oxford UKA (Zimmer Biomet; 20, 23%), Persona Partial Knee (Zimmer Biomet; 10, 11%) and ZUK Unicompartmental Knee (Smith & Nephew; 2, 2%). There were no reoperations for the included patients during the study period.

**Table 1:** Characteristics of patients undergoing primary medial UKA at two tertiary centres between 2017 and 2021.

Patients	
Total, *n*	
Knees	88
Patients	85
Gender	
Male	50 (56.8)
Female	38 (43.2)
Age at surgery	
Mean ± SD	64.1 ± 8.8
Range	46–84
BMI	
Mean ± SD	29.7 ± 5.3
Range	18.0–44.0
ASA status	
1	7 (8.0)
2	62 (70.5)
3	17 (19.3)
4	2 (2.3)
Follow‐up time	
Mean ± SD	3.2 ± 1.1
Range	1.0‐5.4

Abbreviations: ASA, American Society of Anesthesiologists; BMI, body mass index; SD, standard deviation; UKA, unicompartmental knee arthroplasty.

### Outcome measures

The main outcomes measurements were preoperative cartilage loss and patient‐reported clinical outcomes. Preoperative cartilage loss was recorded using the modified ICRS lesion classification from MRI (Supporting Information S1: Table 1) [7] and Kellgren–Lawrence (K–L) scores from radiographs; [17] a higher score representing more severe cartilage loss. For each patient, we also recorded the presence of medial BME. Each of the medial, lateral and PF compartments was assessed for each patient by two radiologists with consensus achieved using a third radiologist where necessary. The medial and lateral compartments of the PF were graded separately. For each patient, the ACL and MCL were also assessed using MRI to ensure they were intact.

Patient‐reported outcome measures were measured using the OKS. OKS was collected preoperatively, and subsequently at 6 weeks, 1 year and 2 years following the procedure. OKS ‘change’ scores were calculated using the difference from baseline for ‘early’ (6 week) and ‘late’ (1‐ or 2‐year follow‐up). For the latter, the scores were combined to form the ‘late’ follow‐up group as minimal change in the OKS is expected between 1 and 2‐year follow‐up [8, 14]. There were 54 OKS responses at the 'early’ follow‐up. For ‘late’ follow‐up, OKS at either 1 year (*n* = 63) or 2‐year follow‐up (*n* = 22) were included for analysis.

### Statistical analysis

Continuous data were analysed and presented as mean ± SDs and categorical data were presented as frequencies (%). Between‐group differences for continuous variables were assessed using *t* tests or 1‐way analysis of variance for normally distributed variables, or Mann–Whitney for nonparametric variables (PRISM 8, GraphPad). Between‐group differences for categorical variables were assessed using *χ* ^2^ or Fisher's exact tests. Groups with fewer than five observations were excluded from further subgroup analyses. For all analyses, a *p* < 0.05 was considered significant.

## RESULTS

### Medial compartment

In the medial compartment, all patients had a preoperative ICRS grade of 4. Out of these patients, 37 (44%) were graded to a radiographic K–L score <4; the majority of patients had preoperative K–L scores of 3 or 4 (80 patients, 95%; Table 2). All but one patient had evidence of medial BME.

**Table 2:** Associations between preoperative radiographic (K‐L) and MRI (ICRS) grading and OKS of the medial and lateral compartments of patients undergoing primary medial unicompartmental knee arthroplasty at two primary tertiary hospitals between 2017 and 2021.

Patients	*n* (%)	OKS change, early mean ± SD	*p* value	OKS change, late mean ± SD	*p* value
Medial compartment					
K‐L score			**0.67** a		**0.12** a
0	1 (1.1)	–		–	
1	1 (1.1)	−9		13	
2	3 (3.4)	−1		19.0 ± 1.4 (18–20)	
3	34 (38.6)	14.0 ± 9.5 (−7 to 28)		18.9 ± 7.5 (2–30)	
4	49 (55.7)	13.0 ± 7.6 (0–29)		21.9 ± 9.1 (−2 to 37)	
Lateral compartment					
K‐L score			**0.72** b		**0.24** b
0	20 (22.7)	13.4 ± 9.4 (−1 to 27)		18.3 ± 9.6 (−2 to 31)	
1	29 (33.0)	11.1 ± 9.6 (−9 to 29)		19.7 ± 8.1 (4–33)	
2	37 (42.0)	13.1 ± 8.4 (−7 to 28)		22.3 ± 8.1 (8–37)	
3	2 (2.3)	18.5 ± 7.8 (13–24)		21.5 ± 3.5 (19–24)	
ICRS score			**0.91**		**0.51**
0	2 (2.3)	8		17.0 ± 5.7 (13–21)	
1	17 (19.3)	12.3 ± 10.4 (−7 to 25)		21.1 ± 8.8 (9–37)	
2	25 (28.4)	13.5 ± 8.4 (0–29)		21.2 ± 7.8 (2–33)	
3	22 (25.0)	13.9 ± 7.9 (0–25)		22.0 ± 6.8 (10–34)	
4	22 (25.0)	11.8 ± 9.9 (−9 to 28)		18.2 ± 10.3 (−2 to 37)	

*Note*: The bold values indicate the significance from the tests.

Abbreviations: ANOVA, analysis of variance; ICRS, International Cartilage Regeneration & Joint Preservation Society; K–L, Kellgren–Lawrence score; MRI, magnetic resonance imaging; OKS, Oxford Knee Score; SD, standard deviation.

^a^ *t* test of K–L grades 3 versus 4.

^b^ ANOVA of K–L grades 0–2.

### Lateral compartment

In the lateral compartment, patients had preoperative ICRS scores from 0 to 4 and K–L scores ranging from 0 to 3. Of those with mild K–L scores (0 and 1, 47 patients), 20 patients (43%) were assessed to have more severe cartilage thickness loss using the ICRS scores (3–4).

### PF compartment

In the PF compartment, patients had preoperative ICRS scores from 0 to 4 for both the medial and lateral facets and K–L scores ranging from 0 to 4 (Table 3). For the medial facet, out of those with mild K‐L scores (0 and 1, 9 patients), seven patients (78%) were assessed to have more severe cartilage thickness loss using the ICRS scores (3–4). For the lateral facet, out of those with mild K–L scores (12 patients), six patients (50%) were assessed to have more severe cartilage thickness loss using the ICRS scores.

### OKS

Preoperatively, patients had a mean OKS of 21.9 ± 6.8 (range 7–36). At early (6‐week follow‐up), the mean OKS was 34.8 ± 8.0 (11–47). At late follow‐up (1–2‐year follow‐up), the mean OKS was 42.4 ± 6.1 (18–48). No associations between K–L or ICRS scores and the OKS were found at early or late follow‐up (Tables 2 and 3).

**Table 3:** Associations between preoperative radiographic (K–L) and MRI (ICRS) grading and OKS of the patellofemoral compartment of patients undergoing primary medial unicompartmental knee arthroplasty at two primary tertiary hospitals between 2017 and 2021.

Patients	*n* (%)	OKS change, early mean ± SD	*p* value	OKS change, late mean ± SD	*p* value
Medial facet, patellofemoral compartment					
K‐L score			**0.32** c		**0.23** c
0	1 (1.6)	–		14	
1	8 (13.1)	4.7 ± 4.9 (−1 to 8)		19.3 ± 7.4 (9–30)	
2	20 (32.8)	11.7 ± 10.6 (−7 to 29)		18.5 ± 8.3 (6–33)	
3	29 (47.5)	13.3 ± 7.9 (0–25)		22.6 ± 8.2 (4–37)	
4	3 (4.9)	14.7 ± 9.1 (8–25)		26.7 ± 11.7 (14–37)	
ICRS score			**0.61**		**0.29**
0	21 (23.9)	10.4 ± 9.7 (−9 to 24)		16.9 ± 8.5 (−2 to 30)	
1	7 (8.0)	13.8 ± 9.5 (0–29)		21.0 ± 7.5 (11–33)	
2	11 (12.5)	13.3 ± 6.3 (8–25)		20.5 ± 6.0 (12–32)	
3	10 (11.4)	18.5 ± 7.9 (12–28)		23.1 ± 7.6 (9–35)	
4	29 (44.3)	12.8 ± 9.2 (−1 to 27)		21.7 ± 9.1 (4–37)	
Lateral facet, patellofemoral compartment					
K‐L score			**0.08** c		**0.12** c
0	1 (1.6)	‐		14	
1	11 (18.0)	6.2 ± 5.6 (−1 to ‐13)		16.0 ± 5.1 (9–21)	
2	34 (55.7)	14.2 ± 9.6 (−7 to ‐28)		22.3 ± 8.6 (4–37)	
3	13 (21.3)	12.3 ± 7.9 (3–29)		20.9 ± 9.1 (8–37)	
4	2 (3.3)	24		28.5 ± 6.4 (24–33)	
ICRS score			**0.37**		**0.87**
0	37 (42.0)	14.4 ± 8.8 (−9 to 29)		20.4 ± 6.9 (9–35)	
1	8 (9.1)	5.5 ± 8.4 (−7 to 11)		17.7 ± 9.9 (2–32)	
2	15 (17.0)	11.4 ± 7.7 (3–25)		20.0 ± 11.8 (−2 to 37)	
3	7 (8.0)	9.3 ± 7.0 (0–‐16)		21.7 ± 9.5 (10–33)	
4	21 (23.9)	13.3 ± 10.1 (−1 to 25)		21.6 ± 8.1 (4–37)	
Average score, patellofemoral compartment					
K‐L score			**0.26** c		**0.14** c
0	1 (1.6)	–		14	
1	4 (6.6)	4.7 ± 4.9 (−1 to 8)		16.7 ± 6.7 (9–21)	
2	22 (36.1)	10.4 ± 9.7 (−7 to 28)		18.7 ± 7.7 (6–31)	
3	30 (49.2)	14.1 ± 8.6 (0–29)		22.3 ± 8.5 (4–37)	
4	4 (6.6)	14.3 ± 8.5 (8–24)		27.0 ± 10.2 (14–37)	
ICRS score			**0.11**		**0.12**
0	12 (13.6)	7.2 ± 8.4 (−9 to 15)		15.6 ± 6.3 (2–27)	
1	22 (25.0)	12.1 ± 7.9 (0–27)		19.5 ± 8.9 (−2 to 35)	
2	22 (25.0)	17.4 ± 9.0 (3–29)		23.7 ± 8.5 (8–37)	
3	21 (23.9)	13.1 ± 10.2 (−1 to 25)		21.1 ± 7.3 (10–34)	
4	11 (12.5)	13.0 ± 7.8 (4–25)		21.1 ± 9.6 (4–37)	

*Note*: The bold values indicate the significance from the tests.

Abbreviations: ANOVA, analysis of variance; ICRS, International Cartilage Regeneration & Joint Preservation Society; K–L, Kellgren–Lawrence score; MRI, magnetic resonance imaging; OKS, Oxford Knee Score; SD, standard deviation.

^c^ *t* test of K–L grades 2 and 3.

## DISCUSSION

The study findings suggest that cartilage thickness loss in UKA patients can be more accurately assessed using MRI compared with standard radiographs alone. The findings support the use of MRI for preoperative assessment of symptomatic patients without a clear bone‐on‐bone diagnosis from radiographs. In this study, 37 (44%) of the medial UKA patients were graded to less severe OA cartilage loss (K–L scores 1–3) using radiographs. However, when the same patients were assessed using MRI, all patients had grade 4 ICRS scores, representing full‐thickness cartilage loss, which is a key criterion for the selection of eligible patients for UKA surgery [10]. Similarly, in both the lateral and PF compartments, several patients with less severe K–L scores (0 and 1) on radiographs had symptoms of more severe cartilage disease with the ICRS classification (3–4) using MRI.

As medial compartment bone‐on‐bone OA is a key indication for UKA, these patients, based on radiographs alone nearly half of our patient cohort, may be considered ineligible for the procedure. However, these patients achieved similar levels of postoperative clinical outcomes compared with those with clear bone‐on‐bone indications on radiographs. This supports the concept that provided there is full‐thickness cartilage loss somewhere in the medial compartment, the primary indication has been met.

In this study, MRI assessment was more accurate for the lateral compartment and similar to the medial compartment. There were no differences in postoperative OKS change scores across OA severity groups. However, it is accepted that evidence of cartilage thickness loss in the lateral compartment is a relative contraindication for performing medial UKA, as these patients may subsequently require revision for OA progression [25]. As revision for OA progression mostly occurs after more than 5 years postsurgery [28], the longer‐term benefits or implications of the improved accuracy are less clear from this study and require longer‐term follow‐up studies to draw definitive conclusions.

There is still some debate on patient suitability when assessing the disease state of the PF compartment. Traditionally, evidence of PF OA was considered a contraindication for UKA, and older UKA series report relatively high numbers of revision cases for PF progression [16, 27]. However, some groups suggest that patients with more severe preoperative wear in the PF joint still benefit from excellent clinical outcomes after medial mobile‐bearing UKA [10, 18]. More recently, studies have also indicated that this extends to fixed‐bearing UKA [5, 16]. In this study, which included mostly fixed‐bearing implants (76%), no associations were found between OKS scores and radiographic evaluations of the PF compartments (either medial or lateral facets), which aligns with the recommendation that evidence of preoperative PF disease is not a definite contraindication for medial UKA, regardless of the bearing design [4, 11, 26].

**Figure 2:**
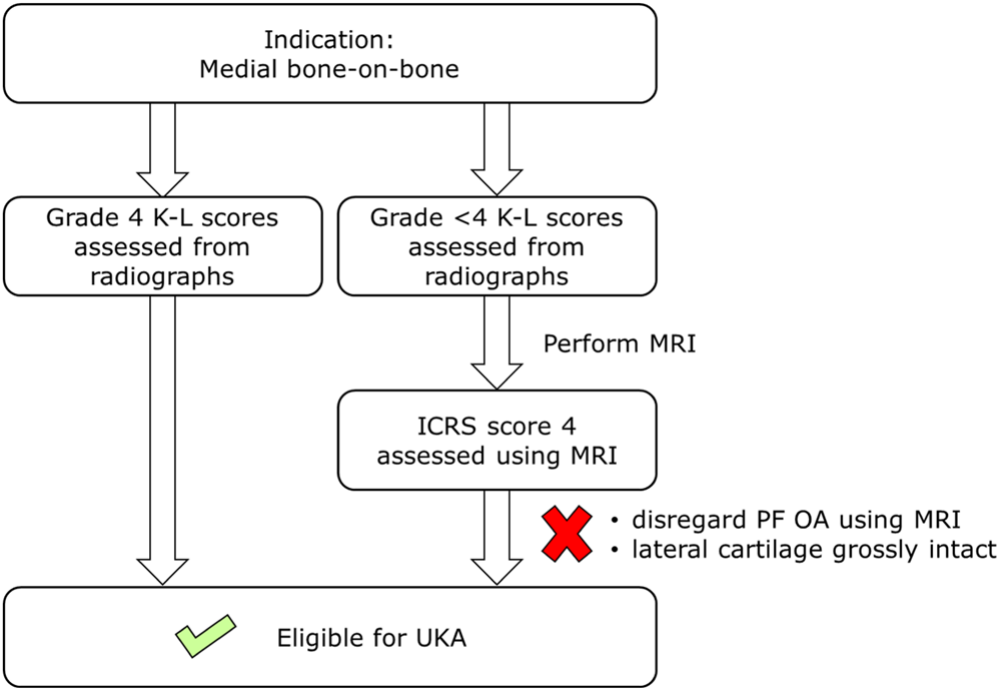
The proposed additional MRI assessment step for improved UKA patient selection. ICRS, International Cartilage Regeneration & Joint Preservation Society; K–L, Kellgren–Lawrence; MRI, magnetic resonance imaging; OA, osteoarthritis; PF, patellofemoral; UKA, unicompartmental knee arthroplasty.

A radiological decision aid has previously been published to assist with patient selection for medial UKA [10]. Based on the findings of our study, an additional MRI step is recommended when assessing the criteria of symptomatic patients without a clear bone‐on‐bone indication on standard radiographs (Figure 2). MRI can be assessed using the ICRS classification for MRI [7].

This study had several limitations. First, this is a retrospective postoperative cohort study. While cartilage thickness measurements between radiographs and MRI could be compared, all patients included in our study had the most severe ICRS score of 4 in the medial compartment. Therefore, these results may be less transferrable to patients with less severe medial OA. However, the patients included in this study are representative of patients undergoing UKA. Second, while it was initially intended to assess the associations of other structural abnormalities detected using MRI, such as medial BME, with postoperative outcomes, all but one of the included patients showed the presence of medial BME, therefore, additional subanalysis could not be performed. Third, while MRI assessment was found to be superior to radiograph assessment, it is possible that some of the radiographs were less optimally aligned. Previous authors have reported that malalignment in radiographs could lead to inaccuracies in assessment [19]. However, we believe that the main study implication is that symptomatic OA patients without evidence of medial compartment radiographic bone‐on‐bone may still benefit from additional assessment using MRI. Fourth, while MRI was shown to be useful in assessing OA severity more accurately in all three compartments, conclusions on the utility of this improved accuracy for the lateral compartment were unable to be drawn as this required long‐term follow‐up of patients to capture revisions due to OA progression in the lateral compartment. This was outside the scope of this study and indicates a potential area for future research. Finally, some early reports suggest that coronal knee alignment of the knee (CPAK) may change with UKA [2, 22, 24], and that preservation of the CPAK phenotype is associated with better clinical outcomes [21, 22, 29]. However, CPAK alignments were not included as a variable for analysis within this study as long‐leg radiographs are not routinely performed for UKA at the two institutions. This could be a potential avenue for future research aimed at optimising clinical outcomes following UKA.

In conclusion, the findings of this study suggest that assessment of cartilage thickness loss using MRI should be used for preoperative assessment of symptomatic patients without clear bone‐on‐bone indication from standard radiographs. For medial UKA patients, MRI should be used for assessment of the medial compartment; however, evidence of disease in the PF compartment assessed using MRI should not be considered a contraindication for UKA.

## AUTHOR CONTRIBUTIONS

A. Paul Monk and David Dow conceptualised the study. Tyler Campbell, Laura Hill, Lydia Lin, Hayley Wong, David Dow, Mei Lin Tay and Scott M. Bolam curated the data. Mei Lin Tay performed formal analysis and data visualisation. All authors participated in data interpretation and investigation. Mei Lin Tay wrote the original draft and all authors took part in reviewing and editing the manuscript.

## CONFLICT OF INTEREST STATEMENT

A. P. M. receives research support and consulting fees from Zimmer, is a board member of ISAKOS and has stock options in FormusLabs. J. T. M. is a paid speaker for Zimmer and Depuy Synthes and receives research travel support from Corin. S. W. Y. receives research support from Stryker and Smith + Nephew, and is a paid consultant for Stryker. The remaining authors declare no conflicts of interest.

## ETHICS STATEMENT

This study was carried out with ethics approval from the Auckland Health Research Ethics Committee (AHREC), AH2545. Patient consent waiver was approved for this retrospective study in line with the current New Zealand National Ethics Advisory Committee (NEAC) standards, due to the sheer number of records, and with appropriate governance approvals in place.

## Supporting information

Supporting information.

## Data Availability

The data that support the findings of this study are not publicly available due to privacy or ethical restrictions.
