# Supplementary material for: Magnetic resonance imaging provides additional utility in the preoperative cartilage assessment of patients undergoing medial unicompartmental knee arthroplasty
Source: Knee Surg Sports Traumatol Arthrosc. 2025 Feb 10;33(6):2214–21. doi: 10.1002/ksa.12611 (PMC12104780; doi:10.1002/ksa.12611)
Supplement: Supplementary file 1 — Supporting information. [file KSA-33-2214-s001.docx]

**Supplementary Table 1:** Modified International Cartilage Regeneration & Joint Preservation Society (ICRS) classification

**Grade 1:** Superficial fissures

**Grade 2:** Lesions <50% of cartilage depth

**Grade 3:** Lesions >50% of cartilage depth

**Grade 4:** Full-thickness lesions or fissures
